# Phage Displayed Peptides/Antibodies Recognizing Growth Factors and Their Tyrosine Kinase Receptors as Tools for Anti-Cancer Therapeutics

**DOI:** 10.3390/ijms13045254

**Published:** 2012-04-24

**Authors:** Roberto Ronca, Patrizia Benzoni, Angela De Luca, Elisabetta Crescini, Patrizia Dell’Era

**Affiliations:** 1 Unit of General Pathology and Immunology, Department of Biomedical Sciences and Biotechnology, viale Europa, 11, 25123 Brescia, Italy; E-Mail: ronca@med.unibs.it; 2 Fibroblast Reprogramming Unit, Department of Biomedical Sciences and Biotechnology, viale Europa, 11, 25123 Brescia, Italy; E-Mails: patrizia.benzoni@med.unibs.it (P.B.); angela.deluca@med.unibs.it (A.L.); elisabetta.crescini@med.unibs.it (E.C.)

**Keywords:** phage display, humanized antibody, growth factors, tyrosine kinase receptors, anticancer therapy

## Abstract

The basic idea of displaying peptides on a phage, introduced by George P. Smith in 1985, was greatly developed and improved by McCafferty and colleagues at the MRC Laboratory of Molecular Biology and, later, by Barbas and colleagues at the Scripps Research Institute. Their approach was dedicated to building a system for the production of antibodies, similar to a naïve B cell repertoire, in order to by-pass the standard hybridoma technology that requires animal immunization. Both groups merged the phage display technology with an antibody library to obtain a huge number of phage variants, each of them carrying a specific antibody ready to bind its target molecule, allowing, later on, rare phage (one in a million) to be isolated by affinity chromatography. Here, we will briefly review the basis of the technology and the therapeutic application of phage-derived bioactive molecules when addressed against key players in tumor development and progression: growth factors and their tyrosine kinase receptors.

## 1. Antibody Display

Antibody molecules are commonly composed of a pair of identical heavy chain (H) and a pair of identical light chain (L) polypeptides, held together by disulfide bridges and non-covalent bonds in a Y shaped form [1]. The antigen-binding site in the molecule, that is located at the two Y tips, is the variable region (V) that diversifies every single molecule and is generated by the combination of *N*-terminal domains of the H and L chains in the so-called fragment antigen-binding (Fab) [2].

**Figure 1:**
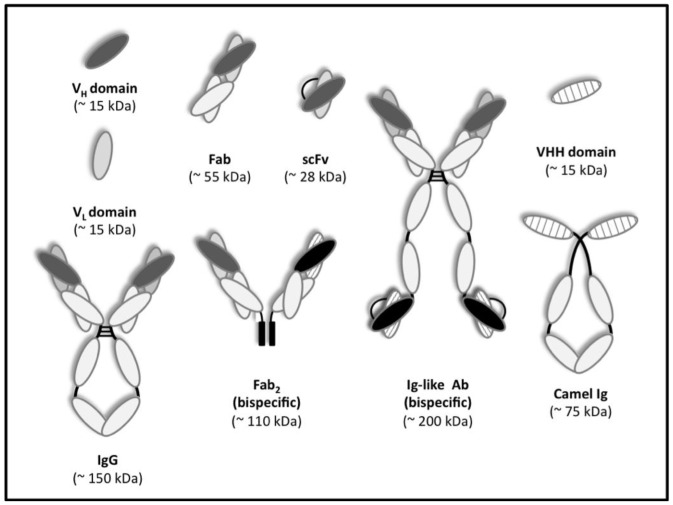
Type of antibody formats, as mentioned in the text, with the relative molecular weight. Adapted from Holliger and Hudson [16].

In 1988, a functional variable region, the immunoglobulin Fv fragment, was produced in *Escherichia coli* [3] and one year later Orlandi *et al*. demonstrated the cloning by PCR of the variable regions of H and L chains, starting from hybridoma cDNA [4]. From this basis came the idea of displaying the antibody library on a phage [5,6] and since then, the genetic information of H and L domains has been transcribed from B lymphocytes or pre-B lymphocytes of several species, PCR amplified, mutated or randomized and subsequently combined by PCR assembly or sequential cloning in the phagemids [7–9]. The filamentous phage commonly used for peptide/antibody display purposes is the single strand DNA virus M13 that presents a rod shaped structure with a circular genome of 6407 nucleotides enclosed in approximately 2700 copies of the major coat protein P8, and capped with 5 copies of minor coat proteins (P9, P6, P3) on the ends [9,10]. The peptides/antibodies of interest are mostly exposed or “displayed” fused to the *N*-terminus of P3 (3–5 copies/phage) or P8 coat proteins (2700 copies/phage) [11]. Despite their abundance on the phage surface, the choice of the protein target depends on the size of the inserted peptide, since long aminoacidic sequences can interfere with physiological phage protein function. Indeed, P8 libraries can display only a six aminoacid peptide in a large number of copies [12], while P3 insertion can tolerate up to 43 amino acids without loss of phage infectivity [13]. However, several advances in engineering M13 coat proteins for improved performances have been made in recent years, thus providing a greater variety of *N*- and *C*-terminal display scaffolds, as well as artificial coat proteins to improve the stability of the genetic information and to increase the number of exogenous peptides exposed on the phage surface [14]. On the other side, several small formats of the antibody molecule that maintain the Fab site have been created and inserted within the phage genome [15]. These antibody fragments, either an entire Fab, a fragment variable (Fv) or a linker-stabilized single chain Fv (scFv), can be displayed by fusion with phage coat proteins [15]. Different antibody formats discussed in this review are shown in Figure 1. The cloning of human repertoires has led to the construction of humanized antibody libraries whose products can be used safely for human diagnostics and therapeutics.

An antibody is used by the immune system to recognize and, eventually, neutralize foreign hosts such as bacteria, viruses, *etc*. Behind the idea of an antibody display is the possibility that the selected molecule will recognize and possibly neutralize the function of the antigen, although the same result could be hypothetically obtained by using a peptide. Nevertheless, the sequencing of the DNA that codify for the aminoacidic sequence of the variable regions of the antibody will allow its transfer, by molecular biology techniques, to various antibody formats, as needed. Challenging the library with the desired human antigen will allow the selection of the binder phages that will be eluted, amplified, and utilized in several cycles of selection, in order to isolate the best candidate for the following purposes. At the end of the procedure, the phage DNA can be sequenced to identify the inserted variable regions, and, by changing the bacterial host, large amounts of soluble antibody fragment or peptide can be produced for further characterization [15].

## 2. Growth Factors and Cancer

**Figure 2:**
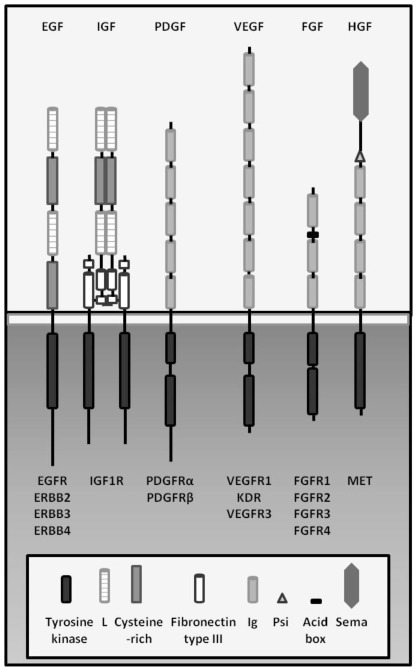
Schematic example of some tyrosine kinase growth factor receptors. Adapted from Lemmon and Schlessinger [17].

Growth factors play a fundamental physiological role in many cell types by driving a wide variety of cellular functions, including growth, differentiation, and angiogenesis. Growth factor signaling involves their binding to high affinity sites on the cellular surface, mostly represented by tyrosine kinase receptor (RTK) molecules. All RTKs have a similar molecular architecture, with ligand-binding domains in the extracellular region, a single transmembrane helix, and a cytoplasmic region that contains the tyrosine kinase (TK) domain plus additional carboxy-terminal and juxtamembrane regulatory regions [17]. Examples of RTKs involved in cell proliferation are shown in Figure 2. Although each growth factor acts in its own way, the common strategy utilized to activate RTK signaling involves the induction of receptor dimerization that, in turn, activates the phosphorylation of specific residues on the receptor intracellular tail. Phosphorylated tyrosines become docking sites for cytoplasmic proteins, whose binding initiate the cascade of events that leads to the activation of transcription factors involved in cell growth, survival, differentiation, or angiogenesis [17].

Cancer cells have defects in regulatory circuits that govern cell proliferation and homeostasis [18]. It has been suggested that cancer is the manifestation of six essential alterations in cellular physiology: self-sufficiency in growth signals, insensitivity to growth-inhibitory signals, evasion of apoptosis, limitless replicative potential, sustained angiogenesis, and tissue invasion and metastasis [18]. Tumor cells sustain their dysregulated proliferation in many ways: in an autocrine manner, by secreting the growth factors themselves and exposing the cognate receptor on cell surface or by inducing host stromal cells to produce the required growth stimulus [19,20]. On the other hand, receptor signaling can be deregulated by enhancing the number of RTKs on the cell surface, thus becoming hyper-responsive to unaltered growth factor availability or by mutating components of the signaling cascade, leading to cell proliferation independently by the presence of receptor ligand [17]. Over-expression and/or structural alteration of RTK family members are often associated to human cancers and tumor cells are known to use RTK transduction pathways to achieve tumor growth, angiogenesis and metastasis. Indeed, recent sequencing efforts in a wide variety of tumors have identified mutations in numerous RTKs, collected in the Catalogue of Somatic Mutations in Cancer (COSMIC) database [21]. A general overview of the association between tumor progression and the altered expression of growth factor/RTK axis is shown in Table 1.

### 2.1. EGF Family/ERBB Family

The ERBB family of RTKs includes the epidermal growth factor receptor (EGFR) ERBB1, also called HER1, and the closely related ERBB2 (HER2/Neu), ERBB3 (HER3), and ERBB4 (HER4) proteins. Aberrant activation by mutation or overexpression of ERBB receptors and their ligands has been found in many human cancers [67]. Indeed, EGFR levels are increased in many epithelial tumors, including head and neck, lung, breast, and colorectal cancers, and its over-expression usually correlates with poor clinical outcome [26,68]. Also, HER2 is frequently altered in human cancers like breast, ovarian, lung, and prostate carcinoma [69] and its up-regulation causes malignant transformation of mammary epithelial cells, breast, ovarian, lung, and prostate carcinoma. Approximately 25% of invasive primary breast cancers exhibit HER2 gene amplification, and this molecular feature correlates with reduced patient survival [70]. Targeting EGFR prevents ligand-induced receptor activation and downstream signaling, resulting in cell cycle arrest, promotion of apoptosis, and inhibition of angiogenesis [71].

### 2.2. HGF/MET

Hepatocyte growth factor (HGF) is expressed by stromal cells while its receptor, HGFR/c-MET, is expressed in a variety of epithelial cells that are involved in organogenesis (lung, kidney, breast) or in neuronal development, muscle development, hematopoiesis and angiogenesis [72]. The HGF-HGFR/c-MET pathway is involved in cancer as well. Indeed, the activation of HGFR/c-MET has been reported to trigger cancer cell proliferation, migration and invasion and to promote tumor vessel angiogenesis, since HGF directly stimulates endothelial cell proliferation and migration [73–75]. Aberrant c-MET activation, including gene amplification, mutation, and overexpression, has been found in hematological malignancies and most solid tumors [76], including gastric, liver, and colon cancers [27,77]. In lung cancer it has been shown that, due to its cross reactivity/compensation effect with EGFR, HGFR/c-MET gene amplification can confer resistance to EGFR-targeting drugs. Thus, the targeting of HGFR/c-MET can potentially be used as a strategy for destroying refractory drug-resistant cancer cells [72].

### 2.3. IGFs/IGFRs

Research and clinical studies have shown that the Insulin Growth Factor 1 Receptor (IGF1R) and its ligands, IGF1 and IGF2, play an essential role, not only in normal growth and development, but also in the development, maintenance, and progression of breast, prostate, and colon cancer [78,79]. IGF mitogenic signaling pathway is an attractive therapeutic target in breast cancer *per se* [80], since high IGF1R levels are associated with resistance to treatment with a monoclonal antibody (mAb) that selectively recognizes the extracellular domain of HER2 and is currently used in the treatment of ERBB2-overexpressing breast cancer [81,82].

### 2.4. VEGFs/VEGFRs

Angiogenesis is a multistep process that results in new blood vessel formation from pre-existing vasculature whose regulation results from a dynamic balance between pro-angiogenic and anti-angiogenic factors [83]. As stated before, a pro-angiogenic switch is strictly required for tumor growth, invasion and metastatic dissemination [84]. Indeed, tumor cells produce growth factors that induce proliferation and migration of endothelial cells, such as Vascular Endothelial Growth Factors (VEGFs), Fibroblast Growth Factors (FGFs), Platelet-Derived Growth Factors (PDGFs) and angiopoietins [85].

The VEGF family of ligands and receptors play a central role in both physiological and pathological angiogenesis, and the development of VEGF antagonists is essential in anti-angiogenesis research [86]. The VEGF family is composed of seven members (VEGF (A–F), PlGF) that act through three structurally homologous tyrosine kinase receptors [VEGFR (1–3)] [87]. VEGF is a homodimeric, basic, 45 kDa glycoprotein, specific for vascular endothelial cells [88] and its binding to VEGFR2/FLK1/KDR causes endothelial cell proliferation, angiogenesis, and increased vessel permeability [89,90]. Anti-angiogenic compounds are postulated both to reduce tumor vascularization, and also to normalize vasculature within the tumor to allow the delivery of anti-tumor drugs [91]. Thus anti-angiogenic drugs specifically targeting VEGF or VEGF receptors (VEGFRs) represent a strategy for tumor control and treatment [92]. Since the introduction of the first mAb approved by the Food and Drug Administration (FDA), humanized bevacizumab (Avastin) that neutralizes VEGF, several drugs targeting VEGF-related pathways have been developed [93]. Also, recombinant antibodies, including scFv fragments, were selected against VEGF or the VEGF-VEGFR complex [94–96].

### 2.5. FGFs/FGFRs

FGFs represent a family of at least 22 structurally homologous polypeptide growth factors that are expressed in almost all tissues. FGFs have been implicated in multiple biological processes during embryo development, wound healing, hematopoiesis, and angiogenesis [97,98]. Among them, FGF1 and FGF2 were identified as angiogenic factors [99,100], promoting the proliferation, migration, differentiation and tubulogenesis of endothelial cells *in vitro* and being potent stimulators of angiogenesis *in vivo* [101], thus playing an important role in tumorigenesis. FGFs interact with a family of four distinct, high affinity RTKs, designated FGFR1/4, whose number is greatly increased by the generation of alternative splicing isoforms of FGFR1, FGFR2 and FGFR3 [102,103]. FGF2, FGFR1, and FGFR2 have been shown to be involved in prostatic cancers [104], non-small cell lung carcinoma [39], and pancreatic cancers [57]. FGFR1 is widely expressed in a variety of tumor-derived cells and tissues and is the major Fibroblast Growth Factor Receptor (FGFR) of vascular endothelial cells [105]. It transduces pro-angiogenic and proliferative signals in human cancers, thus it may represent a target for the development of anti-angiogenic/anti-neoplastic therapies [106,107].

All these observations point to growth factors and their cognate RTK as pivotal targets in cancer therapy approaches. The aim that has been pursued in recent years with phage display libraries is the identification of an antibody or a peptide, recognizing either the growth factor or the receptor that can inhibit their interaction, thus suppressing the resulting proliferative signaling.

Several strategies to block the mitogenic signaling pathway that is activated following ligand-receptor interactions are being evaluated. There are three general classes of agents that inhibit tyrosine kinase receptors: blocking antibodies, small kinase inhibitors, and soluble ligand traps or receptor decoys. To date, agents belonging to all these classes are currently available for therapeutic intervention, and are mainly represented by mAbs directed at the ligand-binding site in the extracellular domain of the receptor and low-molecular-weight inhibitors of intracellular tyrosine kinase activity [108].

## 3. Preclinical Studies

Preclinical approaches using phage display technology are mainly addressed to find and characterize small molecules such as antibodies and peptides with targeting and in some cases neutralizing activity against various members of the growth factors and receptor families. In the last decade almost all the main players involved in tumor growth, angiogenesis, transition processes and all the main steps of cancer progression have been targeted. Obviously, in cancer therapy, the “anti-growth factor approach” addressed to block the ligand-receptor interaction represents a very promising strategy. As already described, growth factors mainly work through their receptors which are plasma membrane embedded proteins generally constituted of three basic parts: the extracellular, the transmembrane and the intracellular regions with functionally and spatially distinct roles. Due to their accessibility and also to their prominent role, receptors represent the largest group of drug targets and most of the therapeutic molecules and strategies are addressed against the extracellular portion/ligand-binding site. The intracellular region is less accessible to peptides or small proteins, which are often too hydrophilic to diffuse across plasma membranes. For this reason only small chemicals, like tyrosine kinase inhibitors, modulate receptor function via an interaction with its intracellular signal-transducing region.

Even though the targeting of a receptor can potentially block all the correlated growth factors, sometimes the physiological interaction with a particular ligand must be preserved. Then, specific growth factors become achievable targets, as they have secreted proteins available in the extracellular space and are often embedded in tumor stroma.

### 3.1. Phages Displaying Peptides

Phage display has been applied to isolate and characterize peptides endowed with anti-growth factor activities. Recently, specific EGFR binding peptides from combinatorial library selection were first screened and then narrowed by structure-function analysis. The most active motif necessary and sufficient for specific EGFR ligand binding, corresponding to EGFR 283–287 (CVRAC) was synthetized as retro-inverted derivative and became the preclinical prototype of choice [108].

Some years before, another ERBB2-avid peptide, discovered from phage display, was evaluated in human breast carcinoma cells and in breast carcinoma-xenografted mice for its potential to be used as a tumor-imaging agent and as a vehicle for specific delivery of radionuclide or cytotoxic agents for tumors overexpressing ERBB2 [109].

A random dodecapeptide library was screened against the fusion protein GST-VEGFR2 (extracellular domains I-IV of VEGFR2 fused to glutathione *S*-transferase). The most active peptide that competed with the VEGF binding to its receptor exerted anti-angiogenic activity both *in vitro* (preventing human endothelial cell proliferation) and *in vivo* (in the chick embryo chorioallantoic membrane) and reduced tumor growth in mice [110]. Another VEGFR-binding peptide was generated by mini peptide display technology and, similar to the previous one, was shown to effectively interfere with tumor growth and metastasis due to its anti-angiogenic effects and to block intracellular signaling pathways involved in tumor progression [111]. Again, phage library-derived heptapeptides [112–114] that completely abolished VEGF binding to cell-displayed VEGFR2, showed the *in vitro* inhibition of VEGF-mediated proliferation of human vascular endothelial cells and a suppression of VEGF-induced angiogenesis in a rabbit corneal model [114].

Also, the FGF2/FGFR pathway has been targeted and inhibited by synthetic peptides. Peptides were selected for their binding affinity to anti-FGF2 antibodies, thus suggesting structural similarity with FGFR. This “mirror” characteristic revealed peptides able to bind and neutralize FGF2. These selected hexapeptides inhibited binding of FGF2 to its high-affinity receptor, and suppressed basal and FGF2-induced proliferation of vascular endothelial cells at submicromolar concentrations [107].

Many other peptides are in preclinical development but, unfortunately, their binding affinities are, in general, too low to support their therapeutic use [115]. This is especially true for antagonistic peptides, which need to occupy at least half of the ligand/receptor interaction site to produce an inhibitory effect. Nevertheless, these targeting peptides can be used to design peptido-mimetic molecules or further improved versions, which are more effective in targeting specific cells or tissues when conjugated with therapeutic or diagnostic agents [116,117].

### 3.2. Phages Displaying Antibodies

The wider class of phage display-derived molecules is certainly represented by antibody fragments, which can be easily exploited for their targeting or neutralizing properties. By the way, neutralizing antibodies do not necessarily recognize the exact receptor-binding epitope of the ligand but may block the ligand-receptor interaction by binding to sites proximal to these regions and sterically prevent their interaction.

There are many examples of anti-growth factor receptor antibodies in the preclinical field, isolated from phage display libraries. The first one, a scFv, has been selected from a library whose repertoire was derived from spleen cells of mice immunized with a soluble form of human VEGFR2/KDR [118]. This antibody fragment was shown to inhibit VEGF-induced KDR phosphorylation and VEGF-stimulated DNA synthesis in human umbilical vein endothelial cells (HUVEC) [118]. Similarly, two other scFvs significantly suppressed the mitogenic response of HUVEC to recombinant human VEGF in a dose-dependent manner and reduced VEGF-dependent cell proliferation by 60% and 40%, respectively. *In vivo* analysis of these recombinant antibodies in a rat cornea angiogenesis model revealed that both antibodies suppressed the development of new corneal vessels [119].

Single-domain antibodies in VHH format, specific for FGFR1, were isolated from a phage-display llama naïve library. Two populations of competitive antibodies were identified and both of them, although recognizing distinct epitopes, specifically labeled receptor-expressing cells in immunofluorescence. Antibodies from both populations effectively prevented FGF-dependent internalization and nuclear accumulation of the receptor in cultured cells [120]. Another scFv antibody (RR-C2) specifically recognized FGFR1α and FGFR1β with a K(d) value of 300 and 144 nmol/L for the two receptor isoforms, respectively [106]. The antibody fragment also recognized FGFR1 when the receptor was exposed on the cell surface, prevented the formation of the ternary complex among FGFR1, its ligand FGF2, and cell surface heparan sulfate proteoglycans, and inhibited FGF2-mediated mitogenic activity in endothelial cells of different origin in a nanomolar range of concentrations [106]. *In vivo*, this anti-FGFR1 scFv hampered the angiogenic activities exerted by FGF2 in the chick embryo chorioallantoic membrane assay and by an FGF-dependent breast cancer cell line in Matrigel assay [106].

Different approaches in this preclinical field include the generation and characterization of stable Ig-like bispecific antibodies (BsAb) that target two antigens at the same time. The BsAb molecule EI-04 was constructed with a scFv against IGF1R attached to the carboxyl-terminus of an IgG against EGFR. EI-04 binds to human EGFR and IGF1R with sub-nanomolar affinity, co-engages the two receptors simultaneously and blocks the binding of their respective ligands with potency similar to the parental mAbs. Because of its double inhibition, an enhanced *in vivo* anti-tumor efficacy over the parental mAbs has been shown in two xenograft models [121]. Indeed, in a targeting/drug delivery perspective some scFvs antibodies were also generated that specifically bind to c-MET protein, as aberrantly expressed c-MET has been implicated in human lung cancer as well as malignancy, metastasis, and drug-resistance in other human cancers. The anti-c-MET scFv showed a selective binding and internalization in several lung cancer cell lines expressing c-MET and *in vivo* fluorescent imaging by scFv-conjugated quantum dots showed higher tumor uptake and increased tumor to normal tissue ratios [122]. In addition, conjugation of scFv with PEGylated liposomes enabled higher delivery of doxorubicin into tumor, thus enhancing its cytotoxic activity [122]. Indeed, several other drugs have been conjugated to scFv in order to enhance their therapeutic potential such as the tubulin polymerization inhibitor monomethylauristatin [123], the potent cytotoxic agent DM1 [124], and alpha-amanitin, a toxin known to inhibit DNA transcription [125].

Regardless of the huge amount of potential candidates, only a restricted number of molecules enter clinical trial screening. In the last decade, phage display has become an interesting and increasing source of such candidates. Despite the availability of the XenoMouse model, a mouse genetically engineered with a “humanized” humoral immune system [126], humanized phage-display technology is the preferred choice of many companies for the ease of use and for the speed of isolation of the molecules of interest [127]. To date, this technology has been used to create at least 35 human mAbs that entered clinical development, mainly applied in oncology or as immunomodulatory drugs.

## 4. Clinical Studies

In the last decade, 45% of total mAb candidates for clinical use were derived from human repertoires and many others are actually in clinical development. So far, several human mAbs have been approved by the FDA for marketing in the United States: adalimumab (Humira; Abbott) [128] and golimumab (Simponi; Centocor) [129], for the treatment of rheumatoid arthritis, psoriatic arthritis, ankylosing spondylitis, Crohn’s disease, moderate-to-severe chronic psoriasis and juvenile idiopathic arthritis; panitumumab (Vectibix; Amgen) to treat EGFR-expressing metastatic colorectal cancer [130]; canakinumab (Ilaris; Novartis), indicated for the treatment of cryopyrin-associated periodic syndromes [131]; ustekinumab (Stelara; Johnson & Johnson), used to treat moderate to severe plaque psoriasis and under investigation for multiple sclerosis, psoriatic arthritis and sarcoidosis [132]; ofatumumab (Arzerra; Genmab), for treating chronic lymphocytic leukemia, refractory to fludarabine/alemtuzumab, and also useful in treating follicular non-Hodgkin’s lymphoma, diffuse large B cell lymphoma, rheumatoid arthritis and relapsing remitting multiple sclerosis [133]; denosumab (Prolia; Amgen), approved for use in postmenopausal women with a risk of osteoporosis and for the prevention of skeletal-related events in patients with bone metastases from solid tumors [134]; and the recently approved belimumab, (Human Genome Sciences) for the treatment of systemic lupus erythematosus [135] and ipilimumab (Bristol-Myers Squibb) for the treatment of melanoma [136], that is also undergoing clinical trials for the treatment of non-small cell lung carcinoma and metastatic hormone-refractory prostate cancer [137]. Moreover, Human Genome Sciences received Biologics License Application from the FDA for raxibacumab, intended for the prophylaxis and treatment of inhaled anthrax [138].

As reported, the field of clinical application for these antibodies is predominantly autoimmunity and inflammatory pathologies followed by cancer therapy. Notably, and despite the central and pleiotropic role of growth factors in cancer biology, only a restricted number of antibodies that entered clinical trials are directed against growth factors and their receptors. For sure, the academic and preclinical effort in growth factor targeting has increased in recent years and many novel candidates are in preclinical validation, waiting for clinical development. Among them, phage-derived compounds represent an emerging category that, in terms of number of candidate identifications, is possibly overcoming other technologies such as chimerization or humanization processes, and XenoMouse platform. As companies mainly own phage display libraries, the preclinical process is often well documented on peer-reviewed original articles while less information is available on the following refinement process performed mainly by private institutions.

Based on these considerations, we tried to provide an overview on the ongoing clinical studies based on human antibodies derived from phage display and conceived as growth factors/receptors targeting agents [139]. To date, some phage derivatives against these classes of targets are in clinical evaluation, among them: IMC-A12, IMC-11F8, IMC1121b and IMC-3C5 (by ImClone), AMG-479 (by Amgen), VGX100 (by Circadian Technologies Ltd). A brief summary of their main properties is reported in Table 2.

### 4.1. IMC-A12 (Cixutumumab)

It is a recombinant fully human IgG1 mAb that specifically targets the human IGF1R. IMC-A12 binds with high affinity to the IGF1R, inhibits ligand-dependent receptor activation and downstream signaling. IMC-A12 also mediates robust internalization and degradation of the IGF1R. Although promising single-agent activity has been observed, the most impressive effects of targeting the IGF1R with IMC-A12 have been noted when this molecule was combined with cytotoxic agents or other targeted therapeutics [140]. In a randomized phase II study IMC-A12 was tested alone and in combination with Cetuximab in patients with metastatic colorectal cancer, refractory to anti-EGFR mAb. In both combinations, results were insufficient to warrant additional study in patients [141]. In 2010, a phase II clinical trial began and is still ongoing for the use of IMC-A12 in patients who had chemotherapy-treated mesothelioma (ClinicalTrials.gov Identifier: NCT01160458).

### 4.2. IMC-11F8 (Necitumumab)

It is a fully human IgG1 mAb against the EGFR with antitumor potency similar to the chimeric Cetuximab/Erbitux and might represent a safer therapeutic alternative [145]. IMC-11F8 specifically blocks the receptor/ligand interaction and is intended for the treatment of solid tumors, such as colorectal cancer, by inducing tumor-specific cell death. IMC-11F8 was evaluated in phase II clinical trial for colorectal cancer therapy and the results were well-tolerated, associated with preliminary evidence of antitumor activity, achieving biologically-relevant concentrations throughout the dosing period [146]. Also, bispecific antibodies obtained, combining IMC-A12 and IMC-11F8, were reported but have not entered the clinic yet [139].

### 4.3. IMC-1121b (Ramucirumab)

It is a human IgG1 mAb that specifically recognizes with high affinity (50 pM) the extracellular VEGF-binding domain of the VEGFR2 [32]. IMC-1121b prevents neo-angiogenesis in solid tumors by inhibiting and blocking VEGFR2, similarly to the FDA-approved antibody bevacizumab (Avastin) that targets VEGF itself. Nevertheless, in contrast to other agents directed against the VEGFR2/VEGF axis, ramucirumab binds a specific epitope on the extracellular domain of VEGFR2, thereby blocking all VEGF ligands from binding to this therapeutically-validated target [147]. IMC-1121b is currently in phase II clinical trial for the treatment of metastatic malignant melanoma, metastatic renal cell carcinoma, and liver cancer. Recently, in a pharmacologic and biologic study of phase I, patients with advanced solid malignancies were treated once a week with escalating doses of ramucirumab. At the end of the study, anti-angiogenic effects and anti-tumor activity were observed over a wide range of dose levels, suggesting that ramucirumab may have a favorable therapeutic index in treating malignancies amenable to VEGFR2 inhibition [32].

### 4.4. IMC-3C5

It is a fully human mAb that specifically binds to and hampers VEGFR3 signaling, thus inhibiting angiogenesis and decreasing tumor nutrient supply [145]. Target selectivity is guaranteed by the fact that VEGFR3 plays a critical role in the embryonic vascular system development but its expression is postnatally restricted to the endothelial cells of lymphatic vessels [148]. Recent studies have shown that VEGFR3 is expressed in many solid and hematologic malignancies, because of its involvement in the tumor-associated lymphangiogenesis process [149,150], making the molecule a strong candidate for anti-tumor therapy. Indeed, a phase I study that began in 2011 will examine the safety and tolerability of escalating doses of IMC-3C5 in patients with advanced solid tumors that are refractory to standard therapy or for which no standard therapy is available (ClinicalTrials.gov Identifier: NCT01288989).

### 4.5. AMG-479 (Ganitumab)

It is an IgG1 mAb that binds to and blocks IGF1R [142], initially characterized for its therapeutic activity on pancreatic carcinoma cells *in vitro* and in xenograft models [142]. Ganitumab successfully demonstrated good pharmacokinetic and pharmacodynamic characteristics in a phase I trial [143] and then entered a small, randomized, placebo-controlled phase II study where the combination with AMG-479/gemcitabine (a nucleoside analog used in chemotherapy) improved overall survival at six months (primary endpoint) and progression-free survival in patients with metastatic pancreatic cancer (Poster Discussion at the 2010 American Society of Clinical Oncology Annual Meeting) [144]. This promising candidate is now moving into phase III testing in this patient population.

### 4.6. VGX-100 (Fresolimumab)

It is a human antibody, developed by Circadian Technologies Ltd. that acts against one of the VEGFR3 ligands: the human VEGFC. As VEGFC has a specific role in causing new lymphatic vessel development, its blockade in tumors may slow cancer metastasis. Treatment for cancers, particularly glioblastoma and metastatic colorectal cancers, are the first target indications for VGX-100. Additionally, Circadian is developing VGX-100 for a number of other cancer indications, as well as an agent to treat front-of the-eye diseases [151]. In 2012 a phase I study will examine the safety and tolerability of escalating doses of VGX-100 in patients with advanced metastatic solid tumors who have no other standard treatment options both as a immunotherapy and also when used in combination with other anti-angiogenic agents such as bevacizumab (ClinicalTrials.gov Identifier: NCT01514123).

By means of phage display technology, the effortless identification of the amino acid sequence of the isolated phage has allowed the *in vitro* construction of several functional antibodies, carrying the identified antigen-binding site to be used as cancer targeting agents. In addition to what we described in this review, two further comments can point up the ongoing beliefs for cancer treatment: the complexity of tumor pathology needs a multitarget approach and, where possible, antibody conjugation. The established idea is that the simultaneous inhibition of angiogenesis, cancer cells and possibly the tumor supporting stroma would gain better results in fighting the disease. To this purpose several antibodies derived from phage display, targeting tumor overexpressed cytokines (e.g., GC-1008, an anti-Transforming Growth Factor β cytokine [152]), tumor stromal antigens (e.g., L19, an anti-isoform B of fibronectin [153]), or tumor-associated antigens (e.g., adecatumumab, that recognize epithelial cell-adhesion molecules [154]) are currently in clinical trials. Furthermore, once the target is really narrowed by the antigen recognition property of the antibody, the conjugation of the latter with cytotoxic or immunomodulatory drugs will strongly increase the therapeutic benefit for patients while reducing systemic drug toxicity [155].

In conclusion, antibody displayed on phage particles has become a milestone technique for the development of humanized therapeutics. Starting from laboratory bench, a lot of promising anti-cancer molecules recognizing growth factors and their TK receptors have been isolated, sequenced, and transferred to various antibody formats up to the bedside of patients. It should be pointed out that the improvements of this technology in combination with the study of the proteome of tumor/cancer cells would greatly increase the number and efficacy of these therapeutic molecules.

## Figures and Tables

**Table 1:** Growth factor and RTK altered expression in different type of tumors.

Tumor type	Growth factor	RTK	References
Glioma/Glioblastoma	FGF2, VEGFC	EGFR	[22–25]
Head-neck squamous cell		Met, FGFR1, FGFR3, EGFR	[26–28]
Esophageal and Gastric	FGF2	Met	[29–31]
Liver/Hepatocarcinoma		Met, VEGR2	[32,33]
Colorectal	FGF2, VEGFA	Met, ErbB2, IGF1R	[34–38]
Lung	FGF2	FGFR1, FGFR2, EGFR	[39–41]
Renal	FGF2	Met, IGF1R, VEGFR2	[32,42–44]
Prostate	FGF2, FGF8	FGFR1, FGFR3, ErbB2, IGF1R	[45–48]
Bladder		FGFR3, ErbB2	[49,50]
Ovarian		FGFR2, ErbB2, IGF1R	[40,51,52]
Breast	FGF8	FGFR1, FGFR2, EGFR, ErbB2, IGF1R	[40,52–55]
Melanoma		FGFR1, FGFR2, VEGFR2	[32,56]
Pancreatic		FGFR1	[57]
Sarcomas		FGFR2, FGFR4, EGFR, IGF1R	[58–61]
Hematological malignancies	FGF2	Met, ErbB2, FGFR1, FGFR3	[52,62–66]

**Table 2:** Brief summary of phage derivatives in clinical evaluation.

Molecule	Company	Commercial name	Target	Ref
IMC-A12	ImClone LLC	Cixutumumab	IGF1R	[140,141]
AMG-479	Amgen	Ganitumab	IGF1R	[142–144]
IMC-11F8	ImClone LLC	Necitumumab	EGFR	[139,145,146]
IMC-1121b	ImClone LLC	Ramucirumab	VEGFR2	[32,147]
IMC-3C5	ImClone LLC	-	VEGFR3	[145,148–150]
VGX-100	Circadian Technologies Ltd	Fresolimumab	VEGFC	[151]
